# Signaling by AWC Olfactory Neurons Is Necessary for *Caenorhabditis elegans*’ Response to Prenol, an Odor Associated with Nematode-Infected Insects

**DOI:** 10.1534/genetics.120.303280

**Published:** 2020-07-17

**Authors:** Tiffany Baiocchi, Kyle Anesko, Nathan Mercado, Heenam Park, Kassandra Kin, Brandon Strickhouser-Monzon, Priscila Robles, Christian Bowman, Han Wang, Paul W. Sternberg, Adler R. Dillman

**Affiliations:** * Department of Nematology, University of California, Riverside, California 92521; † Division of Biology and Biological Engineering, California Institute of Technology, Pasadena, California 91125

**Keywords:** prenol, 3-methyl-2-buten-1-ol, *dcap-1*, *dcap-2*, *clec*-39

## Abstract

Chemosensation plays a role in the behaviors and life cycles of numerous organisms, including nematodes. Many guilds of nematodes exist, ranging from the free-living *Caenorhabditis elegans* to various parasitic species such as entomopathogenic nematodes (EPNs), which are parasites of insects. Despite ecological differences, previous research has shown that both EPNs and *C. elegans* respond to prenol (3-methyl-2-buten-1-ol), an odor associated with EPN infections. However, it is unclear how *C. elegans* responds to prenol. By utilizing natural variation and genetic neuron ablation to investigate the response of *C. elegans* to prenol, we found that the AWC neurons are involved in the detection of prenol and that several genes (including *dcap-1*, *dcap-2*, and *clec*-39) influence response to this odorant. Furthermore, we identified that the response to prenol is mediated by the canonically proposed pathway required for other AWC-sensed attractants. However, upon testing genetically diverse isolates, we found that the response of some strains to prenol differed from their response to isoamyl alcohol, suggesting that the pathways mediating response to these two odorants may be genetically distinct. Further, evaluations leveraging natural variation and genome wide association revealed specific genes that influence nematode behavior and provide a foundation for future studies to better understand the role of prenol in nematode behavioral ecology.

NEMATODES use olfactory cues for many purposes, including finding mates (Simon and Sternberg 2002), avoiding danger (Rankin 2006), and locating food sources (Bargmann 2006; Chalasani *et al.* 2007). In a previous study the odorant prenol (3-methyl-2-buten-1-ol) was identified in association with entomopathogenic nematode (EPN)-parasitized insect cadavers (Baiocchi *et al.* 2017). It has been suggested that prenol may serve as an information cue for EPNs, informing infective juveniles (IJs) that a potential host is not suitable for infection (Baiocchi *et al.* 2017), and as a cue for IJ dispersal from a resource-depleted host (Kin *et al.* 2019). Exploring the genetic basis of the behaviors induced by prenol in EPN species is unfortunately hindered by a lack of molecular and genetic tools.

*C. elegans* dauers are attracted to prenol while EPN IJs are repelled by it. Such differences in response to odors have been previously observed to other odors like farnesol, 2,3-butadione, and CO_2_ (Hallem *et al.* 2011a; Castelletto *et al.* 2014). Despite the differences in behavioral responses, the neuronal and molecular pathways may still overlap, similar to the way *C. elegans* dauers and adults respond differentially to CO_2_ despite utilizing more or less the same neurons and molecular machinery (Hallem *et al.* 2011a,b; Guillermin *et al.* 2017). Prenol might be attractive to *C. elegans* as a food cue since this odor is associated with a variety of plant matter including some fruits (Augusto *et al.* 2000; Ruiz-Bevia *et al.* 2002; Elss *et al.* 2005; Lopes-Lutz *et al.* 2008). This may also explain why *Levipalatum texanum* (Baiocchi *et al.* 2017) and *Pristionchus pacificus* are also both attracted to this odor (Supplemental Material, Figure S1).

The goal of this study was to understand some of the mechanisms that drive *C. elegans* attraction to prenol at the neuronal and genetic levels. The identification of specific genes that have homologs in EPNs may help inform how EPNs sense and respond to prenol. To identify the *C. elegans* genes that influence attraction to prenol, we leveraged the *C. elegans* Natural Diversity Resource (*Ce*NDR) (Cook *et al.* 2016). In addition, we identified the sensory neurons and related molecular machinery involved in the detection of and response to prenol. We also included evaluations of responses to a similarly structured odorant: isoamyl alcohol (IAA), also known as 3-methylbutan-1-ol. IAA is found in a variety of ecological situations relevant to *C. elegans* ecology, including a variety of fruits (Harada *et al.* 1985; Augusto *et al.* 2000), and as byproducts of fermentation by both bacteria (Wright *et al.* 1991) and yeast (Dzialo *et al.* 2017); it is also an odorant that elicits robust attraction in *C. elegans*. It has been demonstrated that IAA is sensed through the AWC neurons (Colbert and Bargmann 1995).

In this study, we evaluated the AWC neurons and numerous other genes purported to be involved with AWC-mediated attraction to odors (including IAA), to determine the involvement of these genes in the detection of prenol by *C. elegans*. Among the genes tested were those in the proposed main signal transduction pathway for odor detection in the AWC neuron. Our study also identified three other genes that are implicated in the response to prenol, including *dcap-2* (and its paralog *dcap-1*), *clec-39*, and other genes that have homologs in EPNs (See Table S2).

## Materials and Methods

### Animal maintenance

Nematode strains were obtained from the *Ce*NDR, the *C. elegans* Genetics Center (CGC), and the National BioResource Project (NBRP) headed by the Mitani laboratory in Japan, as well as strains as described below.

Newly received nematodes were chunked and placed on fresh NGM plates with *Escherichia coli* (OP50) and stored at 21 ± 1° for several days to allow for sufficient growth before freezing. Once the population was large enough they were then frozen, and stored at −80° for long-term storage until they were thawed to create a new culture for use (Brenner 1974).

For propagation and care the *C. elegans* strains were maintained as previously described (Brenner 1974; Sulston and Hodgkin 1988). Briefly, 10 cm NGM plates were seeded with approximately 0.2 ml of OP50 *E. coli* liquid culture, incubated overnight at 37° or for 2 days at room temperature (23 ± 1°), and were used within 3 days to provide a food source for nematodes in culture. The bacterial liquid stock of OP50 was made and stored at 4° (Sulston and Hodgkin 1988; Stiernagle 2006) and was remade as needed (usually within 2–3 weeks). To culture the nematodes, 20–30 adults were placed on NGM plates and were stored either at 17° for continued cultivation, or at 21 ± 1° for use in experiments or freezing. Nematodes for experimental use were generally bleached within 4–6 days after plating. For strains that grew slowly, such as the *dcap-2* knockout mutant (CGC strain number: RB1641; Mitani laboratory at NBRP) allele *tm2470*, cultures were given an additional time to grow (usually 2–4 days) to obtain enough adults to bleach for synchronization of offspring.

### Synchronization via bleaching and experimental preparation

Preparation of a synchronous population of *C. elegans* was done as previously described. Briefly, nematodes were plated onto NGM plates with OP50 as a food source. Populations were then propagated at 20 ± 1° until a sufficient number of well-fed, gravid adults were obtained. Worms were monitored daily to prevent starvation and were subsequently bleached as described (Porta-de-la-Riva *et al.* 2012) to obtain a synchronous population of young adults. After bleaching, nematode eggs were then stored in the 20 ± 1° incubator overnight to hatch, and then the nematodes were stored at 20 ± 1° incubator until the majority of the population were young adults (∼2 days and 8 hr for most strains). At this time, they were used for experiments and were cleaned following the procedure described previously (Margie *et al.* 2013).

### Standard large (10 cm) chemotaxis assay

**Figure 1:**
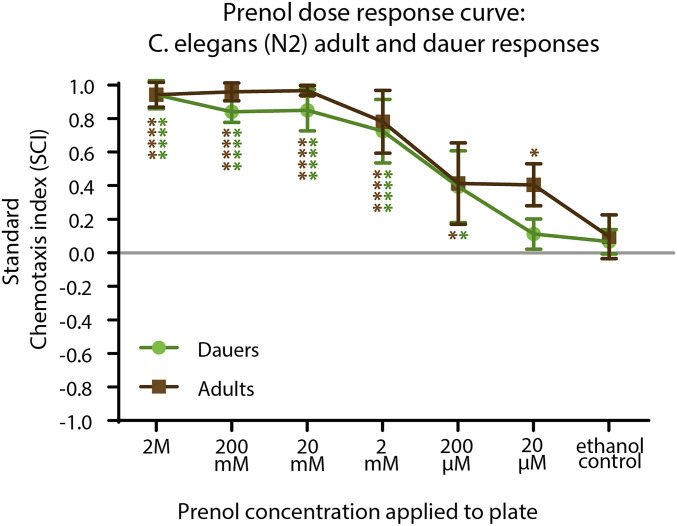
Dose-response curve for *C. elegans* dauers and adults. The concentration of the prenol (in ethanol) dose applied to the test circle of a standard chemotaxis assay. It should be noted that this is the concentration of the prenol solution placed on the plate and not necessarily what the nematodes experienced. Mean is shown and error bars represent SD. Brown asterisks (on left) indicate significant differences between response to doses by adults compared with the ethanol control done for adults. Green asterisks (on right) indicate response to dose by dauers compared with ethanol control dauers. * *P* < 0.05, ** *P* < 0.01, *** *P* < 0.001, **** *P* < 0.0001.

The standard large chemotaxis assay (Figure S3A) (Bargmann *et al.* 1993; Hallem *et al.* 2011a; Baiocchi *et al.* 2017) was used in select areas of this study where evaluating both the standard chemotaxis index (SCI) (Figure S3B) and participation (Figure S3C) was desired. This assay format was used for both the initial dose-response curve evaluation (Figure 1 and Figure S4) and evaluation of *clec-39* (lf) effects on participation (Figure 6). This assay allowed for evaluation of both the SCI as well as participation of the strains that were tested in this manner. The assays were done in the exact same manner as the assays described in “*Behavioral responses of IJs to Chemical odorants*” in Baiocchi *et al.* (2017).

### Quadrant assays

Quadrant assays (Figure S3D) were used for all other behavioral evaluations, and were performed as previously described (Margie *et al.* 2013). In this assay a quadrant template was attached to the bottom on a 6 cm chemotaxis plate. A total of 1 μl of the control (0.5 M sodium azide in water) was then applied on the two quadrants that opposed each other, while 1 μl of the test compound (prenol or IAA diluted into 0.5M sodium azide solution) was applied to the remaining two diametrically opposed quadrants. On the plate ∼200 clean young adults (which had been rinsed three times in S basal buffer) were transferred by pipette to the center of the plate in a 2 μl volume or less. Three plates were then stacked and placed in freezer boxes on top of antivibration platforms. Each experiment consisted of the three plates (technical replicates run in parallel). These assays were conducted at room temperature for 1 hr, and after the allotted time, the nematodes were counted and scored to generate the quadrant chemotaxis index (Figure S3E). Three experiments (biological replicates) were conducted per strain; each experiment (consisting of the three in-parallel, technical replicates) was conducted on different days to account for any effects of day-to-day variations in the laboratory space. The only exception to this was the initial divergent set (Figure 3 and Figure S20) in which six experiments (biological replicates) were done for all strains. For Figure 3, these six experiments were normalized to three experiments to run statistical analysis.

At least 50 nematodes needed to participate (move into one of the quadrants) in the assay in order for it to be entered in the data; however, the number remaining in the center of the plate was not counted (meaning we did not evaluate participation in this assay set up). This rule of a 50 nematode threshold applied to all strains except the *Ce*NDR strain CX2386, in which we observed that consistently <50 nematodes would move into the scoring quadrants regardless of the number of nematodes applied to the plate. For this strain we accepted numbers below the threshold of the 50 nematode minimum and ran a total of six experiments (biological replicates), which were normalized to three experiments to account for the differences and lack of nematodes moving into the scoring quadrants.

A few key differences to note between the large (10 cm) chemotaxis assay and quadrant assay include the size difference, two *vs.* four directions for the nematodes to move, and the placement and method of placement of sodium azide. In the quadrant (6 cm) assay sodium azide is the solute, while in the large chemotaxis (10 cm) assay, 2 µl of sodium azide is placed at the top of the scoring circle immediately before the 5 µl of the control or test compounds are added to the assay arena on the scoring circles. Test chemical is diluted in Milli-Q H_2_O. Aside from these differences, both assays were used to measure the olfactory responses of *C. elegans*. It is important to note that the standard (large 10 cm) chemotaxis assay yields the metric of SCI, similar to the quadrant assays; however, it also provides participation data. Participation data tracks the entire population to determine what percentage of the population moves toward or away from the compounds being tested. The participation helps put the SCI value into context, particularly in whether or not the majority of the population is moving out of the center (initial placement zone). This in turn informs us whether the SCI is representing the majority (over 50%) of the population.

### Drop assays

Chemical drop assays were done as previously described (Hilliard *et al.* 2002). In short, 1-5μL calibrated disposable glass capillary pipets (Drummond Scientific) were used to make needles. Capillaries were pulled using a Sutter Instrument micropipette puller (model P30). Capillaries were pulled into needles using the following settings: heat, 750; pull, 380. Once pulled needles were allowed to sit for at least 5 min before use, the ends of the needle tip were clipped with tweezers and the very end of needles were dipped into the test compound to obtain a small amount of the test compound in the capillary reservoir. The tips of the needs were touched to the agar surface a few times to test that a microdroplet would emerge before nematodes were tested. Each worm that was tested was evaluated for response three times and scored 0 for no responses and +1 for aversion response (stopping, backing up/turning around); the average of 0s and 1s was calculated to determine the nematode’s aversion index score. In total, 80 worms were tested per strain and test compound.

### Touch assays

Touch assays were done as previously described (Hobert *et al.* 1999). In short, an eyebrow or eyelash hair was taped to the end of a Fischer brand 5 and 3/4 disposable glass pasture pipette (13-687-20A) with tape. The hair was dipped in 70% ethanol and dried using a Kimwipe before testing nematodes. Nematodes were touched 10 times, alternating between head and tail, and nematodes were scored a one if they reversed their direction of motion or a 0 if they did not change direction. Response values were then averaged to determine the touch index (proportion of times the nematode responded to touch). This was done for a total of 40 worms for each strain tested.

### Reagents

All chemicals were remade approximately every 4 weeks and stored in glass amber vials within an empty freezer at room temperature to limit light exposure. Sodium azide (CAS 26628-22-8), a paralytic, was obtained from Fisher Chemical (LOT 157679) and is commonly used in chemotaxis experiments (Baiocchi *et al.* 2017; Bargmann *et al.* 1993; Yoshida *et al.* 2012). For quadrant assays, we utilized sodium azide (NaN_3-_) diluted in MQ (Milli-Q) water served as the diluent for most of our odors. This chemical served to paralyze the nematodes (Bargmann *et al.* 1993) after they had made their behavioral decisions in the assay arena; this served to minimize the effects of odor adaptation and to increase the accuracy of quantifying the nematodes in the assay. In these 6 cm quadrant experiments, sodium azide was made at a concentration of 0.5M in MQ water and was also used at a 1M concentration for the large chemotaxis assay.

Prenol (3-methyl-2-buten-1-ol) was obtained from Acros Organics (LOT A0360271, CAS 556-82-1). Prenol was diluted to a concentration of 20 mM in 0.5M sodium azide, while in large chemotaxis assays prenol was made at a concentration of 20 mM in MQ water.

IAA (3-methyl-1-butanol; CAS 123-51-3) was obtained from Tokyo Chemical Industry America and was used as a positive control. In a 6 cm quadrat experiment, IAA was diluted to a concentration of 20 mM in 0.5M sodium azide.

For 0.1% SDS, a stock of 1% SDS (weight by volume), using Fisher brand SDS micropellets (BP8200-500), was prepared using sterile Milli-Q water, placed on a rocker overnight to dissolve at room temperature, and held at room temperature. Preparations of (a 1:10 dilution) 0.1% SDS were prepared freshly each time before use.

Sigma-Aldrich brand octanol (472328-100ML) was used for experiments. Octanol was held at room temperature and a 20 μl aliquot was pulled from the stock bottle for each experiment.

### Generation of knockout mutants using CRISPR/Cas9

New putative null mutants of candidate genes from this study were generated using a coconversion CRISPR/Cas9 strategy, the STOP-IN method (Wang *et al.* 2018). Briefly, preassembled Cas9 ribonucleoprotein and short single-stranded DNA oligo repair templates were injected to the gonad of the wild-type N2 strain, according to the standard microinjection protocol for *C. elegans* (Mello and Fire 1995). Desired mutants were identified in following generations by PCR. All alleles were confirmed by Sanger sequencing. The sequences of guide RNAs and repair oligos, as well as the allele information of the resultant strains, are listed in Table S21.

### Generation of rescue lines

Rescue lines were generated using the *dcap-2** lf* mutant (*ok2023*) (CGC strain: RB1641). To rescue the phenotype, the plasmid construct pHW554(P*ceh-36*::*dcap-2a cDNA*::*let-858* 3′UTR) was constructed and microinjected into adult *C. elegans* with a P*myo*-2::NLS::GFP co-injection marker. Additional details are available in the resource summary Table S21.

### Statistical analyses

For dose responses (Figure 1A), each data point comprises three experiments (biological replicates), and each experiment is made up of three plates run in parallel. Comparisons between responses to dose and responses to the ethanol control were evaluated use GraphPad PRISM software, utilizing and two-way ANOVA with Dunnett’s correction for multiple comparisons post-test. Statistical analysis of participation (Figure S4) was done using a two-way ANOVA, with Sidak’s multiple comparisons test, comparing adults and dauers for each dose tested.

**Figure 2:**
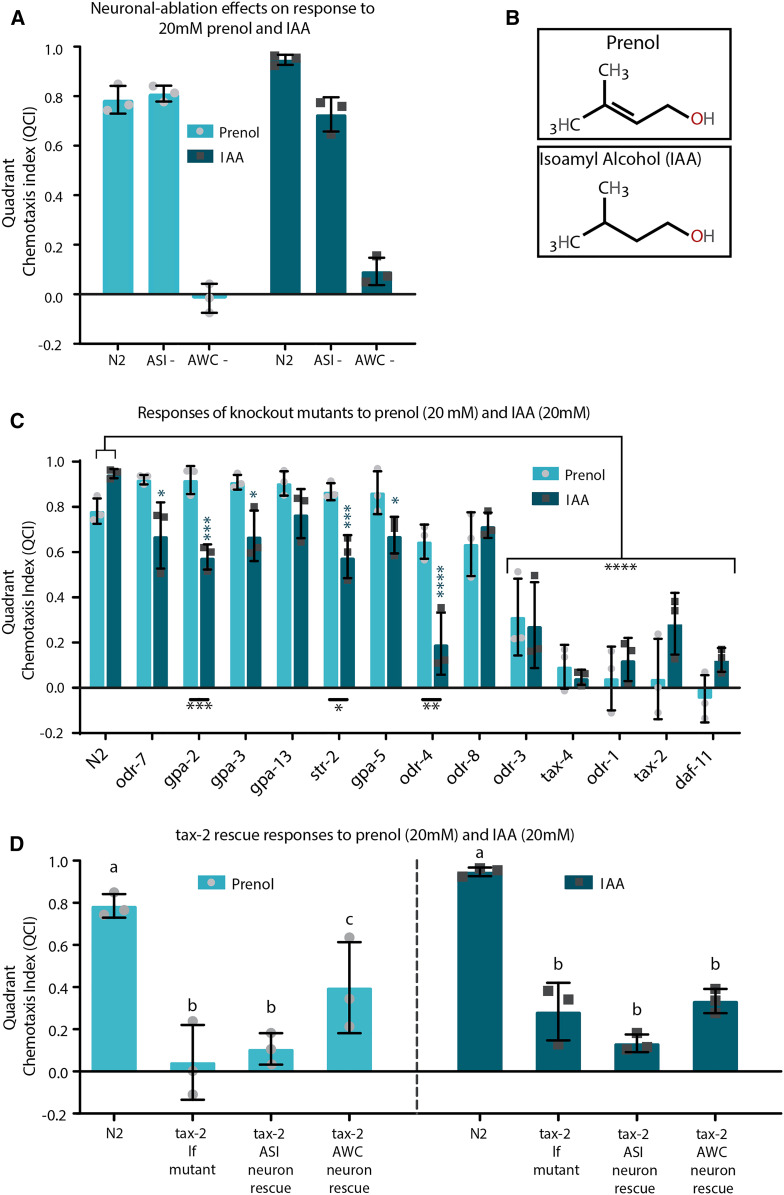
Evaluation of neuron-ablated strains and gene knockout mutants. (A) Responses to 20 mM IAA and 20 mM prenol by *C. elegans* strains with specific neurons genetically ablated. On the left (light teal bars with light gray circles representing data points) is responses to 20 mM prenol by N2 (our wild-type control), ASI− (PY7505 *oyls84*) in which the ASI neurons have been genetically ablated), and AWC− (PY7502 *oyls85*) in which the AWC neurons have been genetically ablated. On the right (dark teal with dark gray boxes representing data points) are these same three strains, evaluated for their response to IAA (20mM). ** *P* < 0.01, **** *P* < 0.0001. (B) Illustration showing prenol and isoamyl alcohol, to show that these two chemical compounds are similar in structure. (C) Responses to 20 mM prenol (light teal) and 20 mM isoamyl alcohol (dark teal) by various genetic knockout (*lf*) mutants, most of which were selected based on the presence in the AWC neurons. For *odr-3*, *tax-4*, *odr-1*, *tax-2*, and *daf-11* the responses to both prenol and IAA were significantly different from either of the responses to IAA and prenol by N2 (indicated by black lines and asterisks above data bars). Other statistically significant differences between IAA responses of other *lf* mutants compared to N2 are designated by the light blue asterisks above the data bars. Results of comparisons between prenol and IAA responses across all *lf* mutants revealed significant differences between responses to IAA and prenol for the *lf mutants* of *gpa-2*, *str-2*, and *odr-4*, as designed by black asterisks below the data bars. (D) Responses to prenol (20 mM) and IAA (20 mM) by N2, *tax-2** lf* mutant (p671), and *tax-2*-rescue lines expressing *tax-2* in either the AWC neuron (ZC2584) or the ASI neuron (ZC2691) (both rescues used the *tax-2* (lf) allele *p691*). Significant differences were seen between responses of N2 and other strains for both odors, but for response to prenol we note that only a partial rescue was achieved in the AWC neuron rescue line, which exhibited significantly increased attraction compared to the original *tax-2** lf* mutant as well as the ASI rescue. For more information on strain designations for any mutant see Table S21 (resources summary). For all graphs the mean is shown by the bar height and error bars represent SD, light gray circles (for prenol) and dark gray boxes (for IAA) indicate individual data points; each data point represents the average value for three in-parallel (technical) replicates ran on the same day for a total of nine experiments per strain. ** *P* < 0.01, **** *P* < 0.0001.

For evaluation of *C. elegans* strains with specific neurons genetically ablated and their responses to prenol and IAA (Figure 2A), a two-way ANOVA was used with Sidak’s multiple comparisons test to compare between N2, AWC−, and ASI− within each category of prenol and IAA responses (but not between responses of IAA and prenol). Comparing the responses between N2 and all other strains (for both IAA and prenol) was done using and two-way ANOVA with Dunnett’s multiple comparisons test; comparing prenol responses to IAA responses across all strains utilized a two-way ANOVA with Sidak’s multiple comparisons post-test (strictly comparing IAA to prenol across all strains evaluated). For comparing the *tax-2* (lf) mutant and rescue lines (Figure 2C), a two-way ANOVA with Sidak’s post-test was used comparing responses to either prenol or IAA across all strains evaluated.

**Figure 3:**
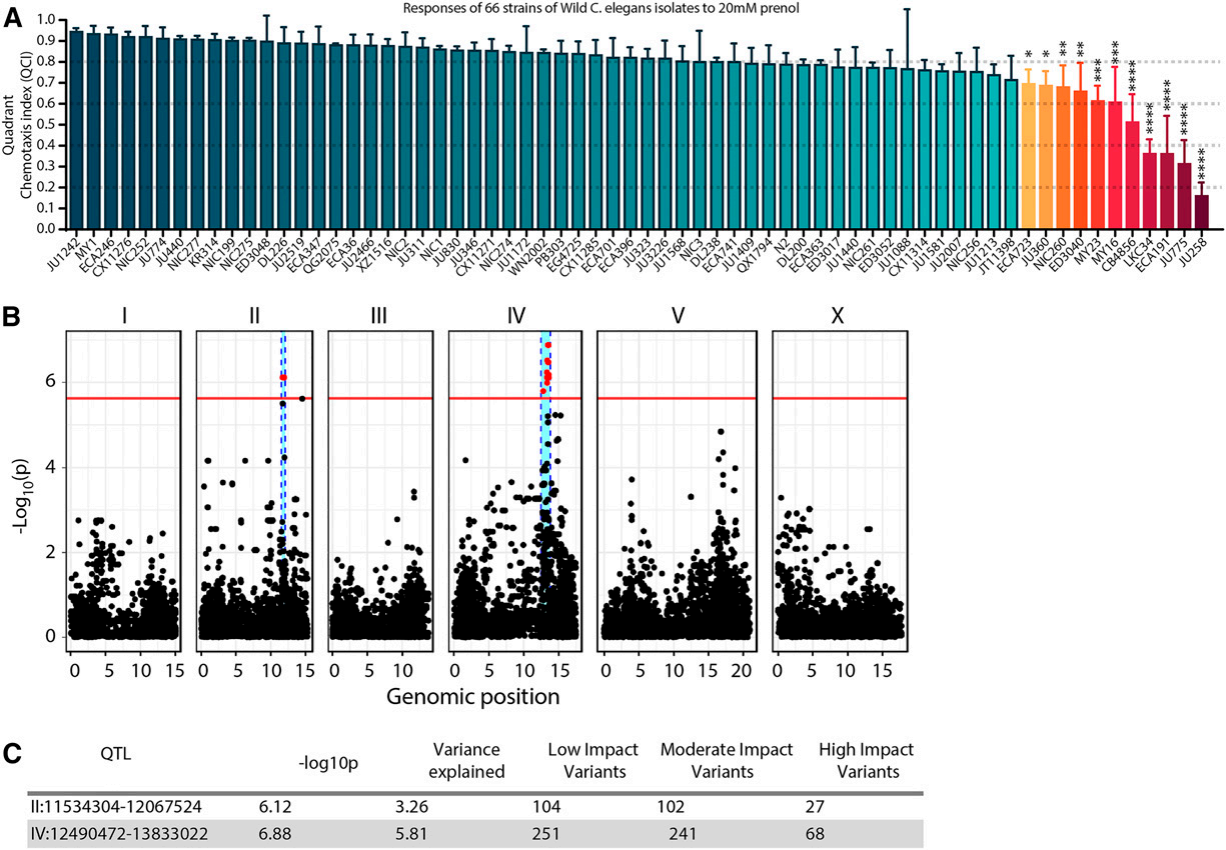
Results from *Ce*NDR strains and analysis. (A) Results of chemotaxis assays evaluating responses to prenol by wild isolates of *C. elegans*. Most strains displayed strong attraction to prenol with 11 of the 66 strains exhibiting significantly less attraction compared to the top scoring strain (J1242). Mean is shown and error bars represent SD. * *P* < 0.05, ** *P* < 0.01, *** *P* < 0.001, **** *P* < 0.0001. (B) Manhattan plot based on Quadrant chemotaxis index (QCI) values exhibited by *Ce*NDR strains tested. Along the *x*-axis the genomic position for each chromosome is displayed and along the *y*-axis the levels of significance for the association tested performed by *Ce*NDR is displayed. The red bar set at ∼5.5 was set by the Bonferroni-corrected value of $\left(-\log\;\frac{0.05}{\mathrm{no}.\;\mathrm{of}\;\mathrm{SNVs}}\;\right)$ . The red dots indicate single nucleotide variants (SNVs) that display significance values higher than the threshold. The blue highlighted regions within chromosomes II and IV are the quantitative trait loci identified by the analysis; the QTL region is defined by identifying significant SNVs along with the 50 SNVs before and after the last significant SNV. (C) A breakdown of each QTL displayed in B, displaying the location and number of low-, moderate,- and high-impact variants for protein-coding genes within each QTL. The variance explained by each QTL has also been reported as well as the −log10 (*P*) values as were identified by *Ce*NDR. For more information on strain designations for any mutant see Table S21 (resources summary).

Statistical analysis of the *Ce*NDR results (Figure 3A), and its components (the divergent set, shown in Figure S20) were evaluated using one-way ANOVA with Sidak’s multiple comparisons post-test. For the full set (of the divergent set, mapping set 1, and alternative strains) shown in Figure 3A, all strains were compared against JU1242 (the top scoring strain). For the divergent set (Figure S20), all comparisons were against N2 as a representative of a high-scoring strait. Additionally, in the *Results* and *Discussion* below, a comparison with N2 is also mentioned regarding the full compilation of all strains (Figure 3A); this too was done using a one-way ANOVA with Sidak’s multiple comparisons post-test.

**Figure 4:**
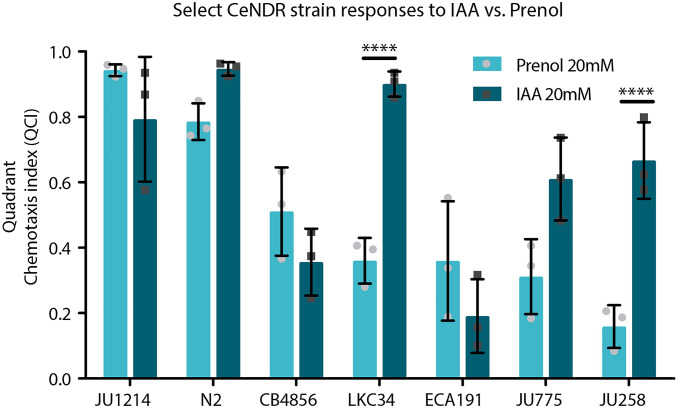
Select *Ce*NDR strain responses to IAA (20 mM) *vs.* prenol (20 mM). A comparison between responses to 20 mM IAA and 20 mM prenol among indicated strains, including five wild isolates that exhibited the most significant reduction in attraction to prenol. Mean is shown by the bar height and error bars represent SD. Light gray circles (for prenol) and dark gray boxes (for IAA) indicate individual data points; each data point represents the average value for three in-parallel (technical) replicates ran on the same day for a total of nine experiments for most strains (with the exception of LKC34, JU775, and JU258 prenol responses, which were normalized from a total of 18 replicates). * *P* < 0.05, **** *P* < 0.0001.

In the prenol *vs.* IAA comparisons for select *Ce*NDR strains, shown in Figure 4, the data were analyzed using a two-way ANOVA with Sidak’s multiple comparisons post-test, comparing IAA and prenol response for each strain. Data related to this is presented in Figure S10, and was analyzed using a one-way ANOVA with Sidak’s multiple comparisons post-test to compare the responses to IAA among all the strains (comparing every strain to every other strain).

**Figure 5:**
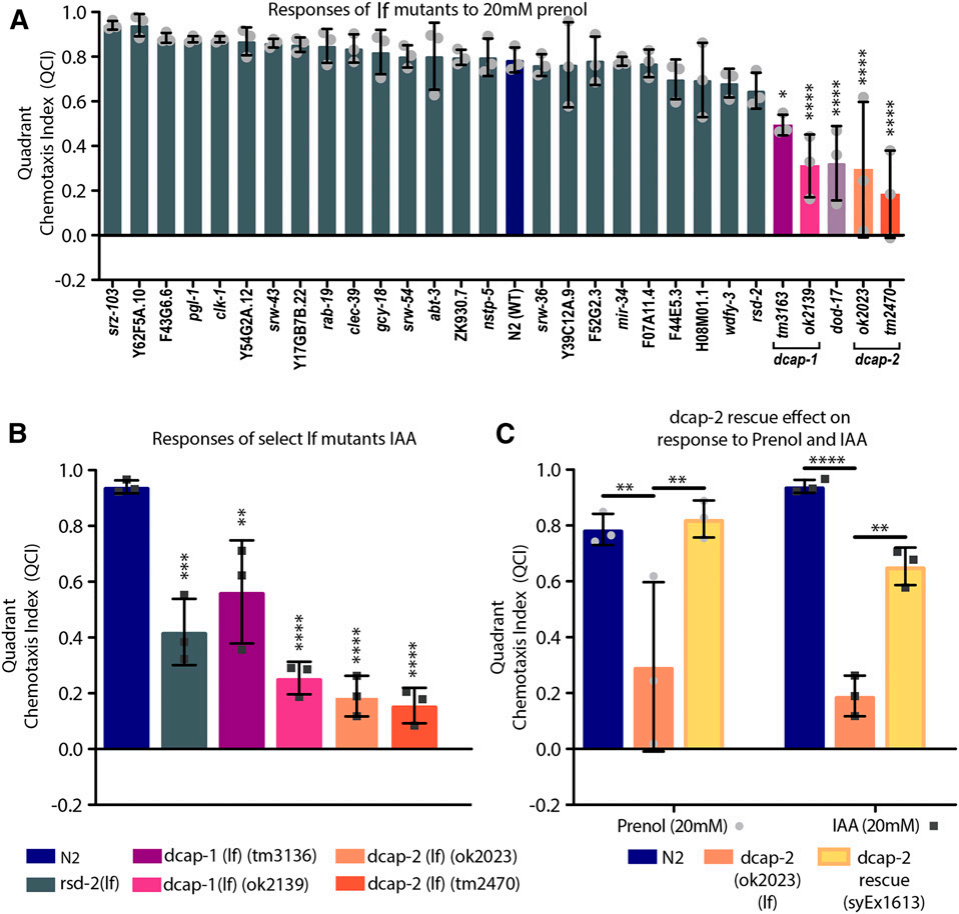
Responses of loss of function (*lf*) mutants to prenol and isoamyl alcohol (IAA). (A) *lf* mutants were selected after analysis of *Ce*NDR results. The *x*-axis displays the gene that was knocked out to create each *lf* mutant. The genes for which we tested multiple alleles but no effect on attraction to prenol were found (gray bars), and only one representative allele is shown. Designations are as follows “gene (allele)”: *srz-103* (*sy1254*), Y62F5A.10 (*sy1222*), *srw-43* (*sy1241*), *srw-54* (*sy1235*), *srw-36* (*sy1249*), Y39C12A.9 (*sy1231*), F52G2.3 (*sy1233*), F44E5.3 (*sy1225*), *rsd-2* (*pk3307*), and *dod-17* (*ok2387*). Figure S11 shows the full set of all strains/alleles tested for all genes evaluated. For additional details on these alleles listed above see Table S21 (resource summary). A one-way ANOVA with Dunnett’s multiple comparisons post-test was used to compare all *lf* mutants against N2 (blue bar). Results revealed three low-response *lf* mutants: *dcap-1*, *dcap-2*, and *dod-17* (*ok2387*) (bars of these *lf* mutants are highlighted in magenta to orange). Please note that evaluations of additional *dod-17** lf* mutants revealed that this gene does not actually influence response to prenol (Figure S12). (B) Responses to IAA by the five lowest-responding mutants (not including *dod-17*): *rsd-2* (*tm1429*), *dcap-1* (*tm3163* and *ok2139*), and *dcap-2* (*ok2023* and *tm2470*). For more information on strain designations for any mutant see Table S21 (resources summary). (C) Comparisons of N2, the *dcap-2* (*ok2023*) *lf* mutant, and the *dcap-2** lf* rescue (*syEx1613*) for responses to both IAA (20mM) and prenol (20mM). For all graphs, the mean is shown by the bar height and error bars represent SD. Light gray circles (for prenol) and dark gray boxes (for IAA) indicate individual data points; each data point represents the average value for three in-parallel (technical) replicates ran on the same day for a total of nine experiments per strain. Mean is shown and error bars represent SEM. * *P* < 0.05, ** *P* < 0.01, *** *P* < 0.001, **** *P* < 0.0001.

For evaluation of gene candidates from *Ce*NDR (Figure 5A), a one-way ANOVA with Sidak’s post-test was used to compare all designated strains (alleles) as described in the figure legend to the responses by N2. Figure S11 contains all alleles that were evaluated in this study, these data were analyzed similarly to Figure 5A, using a one-way ANOVA with Sidak’s multiple comparisons test, comparing the responses of all strains to those of N2.

For the comparisons of *dcap-1*, *dcap-2*, *rsd-2*, and N2 (Figure 5B), a two-way ANOVA with Sidak’s multiple comparisons post-test was used to compare the IAA responses of loss-of-function mutants to those by N2. For analysis of the *dcap-2* rescue (Figure 5C), a representative rescue line was compared to N2 and the *dcap-2* (lf) mutant (*ok2023*; strain: RB1641 from the CGC, same allele/strain used to create the rescue line). This was done using a two-way ANOVA with Sidak’s multiple comparisons post-test to compare the response of each strain to every other strain within the odorant category of prenol or IAA (but not between the odor categories). Response of all rescue lines were evaluated and compared to N2 and the *dcap-2* mutant (*ok2023*), as is shown in Figure S14, which likewise utilized a two-way ANOVA with Sidak’s multiple comparisons post-test comparing all evaluated strains to each other within the category of either response to prenol or response to IAA (but not between the two odors).

**Figure 6:**
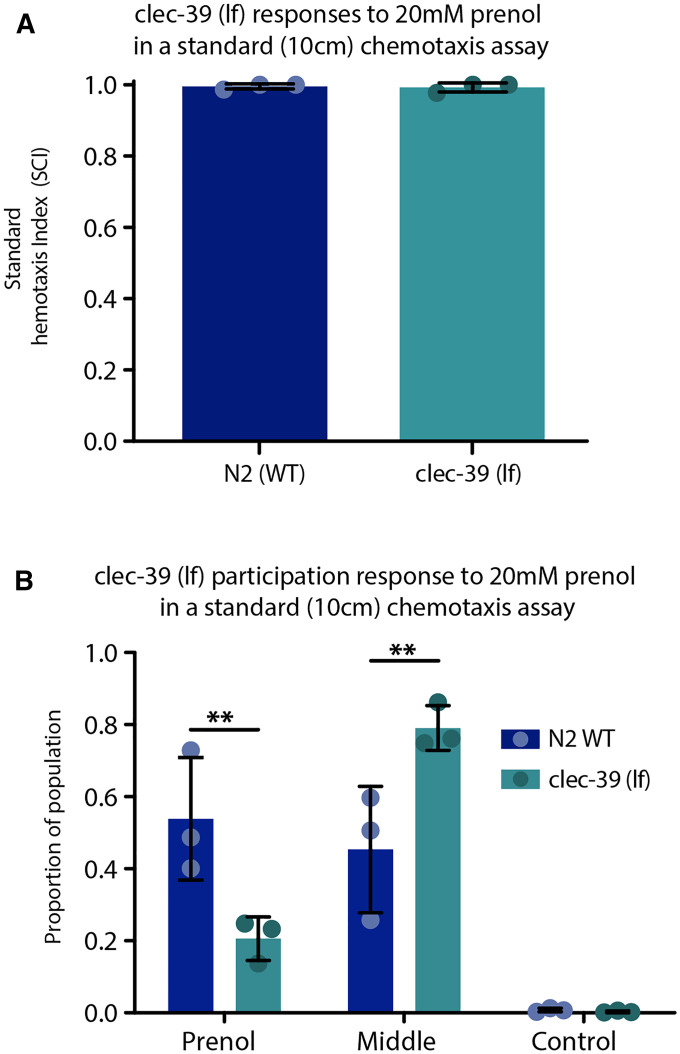
Standard chemotaxis index (SCI) and participation scores for *clec-39* mutants in response to prenol. (A) SCI responses for *clec-39* revealed strong attraction to prenol, comparable to N2 responses. (B) Participation measures reveal that *clec-39* knockout mutants exhibit far less participation in response to the attractive odor prenol. For both graphs the mean is shown by the bar height and error bars represent SD, light blue circles (for N2) and dark green circles (for *clec-39*) indicate individual data points; each data points represents the average value for three in-parallel (technical) replicates ran on the same day for a total of nine experiments per strain. ** *P* < 0.01.

In Figure 6A, a two-tailed, paired *t*-test was used to compare the SCI values between the *clec-39** lf* mutant and N2. Participation scores (in Figure 6B) were statistically analyzed using a two-way ANOVA with Sidak’s multiple comparisons post-test comparing N2 to the *clec-39** lf* mutant for each category of “to prenol”, “middle,” and “to control,” but not between these categories.

Evaluation of *P. pacificus* *vs.* *C. elegans* responses to prenol (Figure S1) was analyzed using a two-way ANOVA with Sidak’s multiple comparisons post-test, comparing all conditions and species to species.

For *C. elegans* adult and dauer participation results in response to varying doses of prenol (Figure S4) statistical analysis was done using a two-way ANOVA with Sidak’s multiple comparisons test, comparing responses between adults and dauers for each dose tested. This was done for each response category of “toward prenol” (in panel A), “middle” (in panel B), and “to control (in panel C).

Evaluation of *dod-17* alleles (Figure S12) was statistically analyzed using a two-way ANOVA with Sidak’s multiple comparisons post-test, comparing responses between prenol and IAA only.

Evaluation of individual *rsd-2* alleles (Figure S13) was analyzed using a two-way ANOVA with Tukey’s multiple comparisons post-test, comparing only within the categories of either response to prenol or IAA but not between the conditions.

Evaluation of individual *dcap-2* rescue lines (Figure S14) was analyzed by a two-way ANOVA with Tukey’s multiple comparisons test, evaluating responses within either prenol or IAA treatment (but not between these conditions).

Each of the drop assays performed (Figure S17) (Milli-Q water, 0.1% SDS, and octanol) was statistically analyzed using a one-way ANOVA with Tukey’s multiple comparisons post-test to compare the responses of all strains to one another. Likewise, all of the touch assays performed (head, tail, and overall results) were statistically analyzed using a one-way ANOVA with Tukey’s multiple comparisons post-test comparing all strains to one another.

Additional responses of *Ce*NDR strains to 20 mM prenol (Figure S22B) was analyzed using a one-way ANOVA with Dunnett’s multiple comparisons post-test, comparing all strains to the top-scoring strain JU1242.

Selected ALT strain comparison to N2 and (lf) mutants (Figure S23) were statistically analyzed as follows: panel A: two-way ANOVA with Tukey’s multiple comparisons post-test comparing all strains to one another within the category of either response to prenol or response to IAA (but not between these treatments); panel B: one-way ANOVA with Tukey’s multiple comparisons post-test, comparing all strains to one another; and panel C: two-way ANOVA with Tukey’s multiple comparison post-test, comparing all strains to one another within the category of either response to prenol or response to IAA (but not between these treatments).

### Data availability

All reagents and strains are available upon request. All relevant data are listed within the paper and within the supplementary materials. Raw data and other information are available from the corresponding author upon reasonable request. Nematode strains are listed in Table S21. An analysis of high-impact-variant genes with AWC expression is available in Table S19. The homology/ortholog analysis of high-impact-variant genes is available in Table S2. Supplemental material available at figshare: https://doi.org/10.25386/genetics.12616691.

## Results

### Characterization of the *C. elegans* response to prenol

We evaluated the ability of *C. elegans* to detect and respond to prenol by performing a dose-response experiment using a standard chemotaxis assay (Figure S3A). In a previous study (Baiocchi *et al.* 2017) nematode behavioral responses to prenol were characterized using EPN IJs, and subsequently *C. elegans* dauers (equivalent stage to IJs). To yield comparable data, we first evaluated the dauer chemotaxis response (as calculated using the equation depicted in Figure S3B) to several concentrations of prenol (Figure 1), followed by evaluation of young adult chemotaxis response to various doses of prenol. We found that *C. elegans* adults and dauers are attracted to prenol, with dauers responding to concentrations as low as 200 µM and adults responding to concentrations as low as 20 µM (Figure 1). In addition, adults had higher participation (calculated using equation depicted in Figure S3C) compared to dauers, meaning that a larger proportion of the population participated in the assay. For some nematode species, participation can be quite low, indicating that the SCI value is not representative of the majority of the population (Baiocchi *et al.* 2017). *C. elegans* young adults appeared to exhibit consistently high participation (∼50%) across all doses tested, while dauers appeared to have participation of <50% at the 200 µM dose or below (Figure S4).

### AWC neurons and canonical AWC signaling pathway mediate response to prenol

In the process of identifying the molecular basis of responses to prenol we explored the role of specific neurons. Comparison between N2 (wild-type control) and strains in which either ASI or AWC neurons had been ablated revealed that removal of ASI has no notable effects on the response to prenol, while removal of the AWC completely eliminated attraction to prenol (Figure 2A). As a control, responses to IAA, which has a structure similar to prenol (Figure 2B) and is known to be detected by AWC, were also tested using these strains. Consistent with previous studies (Bargmann *et al.* 1993; Coburn and Bargmann 1996; Komatsu *et al.* 1996; Wes and Bargmann 2001), without the AWC neurons, attraction to IAA was drastically reduced (Figure 2A). The removal of the ASI also led to a small, but significant reduction in attraction to IAA compared to the attraction seen for N2 (Figure 2A).

Having identified the necessity of AWC for detection and response to prenol, several (*lf*) mutants were evaluated to identify signal-transduction pathway genes that are involved in the detection of or response to prenol as well as IAA. We found that detection of prenol likely utilizes the canonical pathway identified for most attractive odors that are sensed by AWC. The process is predicted to involve an unknown GPCR, DAF-11, ODR-1, ODR-3, TAX-2, and TAX-4, among other proteins and secondary messengers (Bargmann 2006). Loss of function in any one of these signal transduction genes listed above—*daf-11* (*m47*), *odr-1* (*n1936*), *tax-2* (*p671*), or *tax-4* (*p678*)—resulted in a significant reduction or elimination of attraction to prenol (Figure 2C). We also note that among the other genes tested, three of these (lf) mutants (*gpa-2*, *str-2*, and *odr-4*) displayed significantly higher attraction to prenol than IAA.

In addition to the AWC-related genes, we included evaluation of ODR-7 (Figure 2C), a nuclear hormone receptor required for the AWA neurons to express olfactory signaling molecules (Sengupta *et al.* 1994). We reasoned that if this loss of function mutant exhibited reduced attraction to prenol, this would indicate that the AWA neuron and related genes may be involved. However, the *odr-7* (*lf*) mutant had no defect in response to prenol. While ablation of AWA would be a more direct test, the fact that *odr-7* (*lf*) had no effect on the response to prenol suggests that the AWA neuron is not necessary for the detection of prenol.

Lastly, rescue lines of *tax-2* under neuron-specific promotors yielded a partial recovery of attraction behavior when *tax-2* was expressed under an AWC-specific promotor, but this effect was only observed in response to prenol (Figure 2D).

### *C. elegans* wild isolates vary in their response to prenol

The genes we identified above are known to serve in the canonical pathway for detecting multiple odors, including IAA, butanone, and benzaldehyde (Bargmann 2006; Bargmann *et al.* 1993). To further our investigation into the genetic components that either drive or influence attraction to prenol, we utilized the *Ce*NDR, a genome-wide association tool to identify genetic loci associated with various phenotypes. We evaluated 66 natural isolates, 11 of which exhibited significantly reduced attraction to prenol compared to the most attracted natural isolate (Figure 3A). Comparing genetic variation between these strains revealed two quantitative trait loci (QTL); one on chromosome II and one on chromosome IV (Figure 3B). The QTL on chromosome II spanned 0.543 Mb, while the QTL on chromosome IV spanned just over 1.34 Mb. Within these two QTL we found 95 genes with high-impact variants (variants that would likely result in loss of function such as a frameshift or a premature stop codon), which became our initial list of additional gene candidates. The results also revealed a list of 344 genes with moderate-impact variants, which included missense mutations, splice region variants, and in-frame deletions and insertions. Although missense and splice region mutations have previously been shown to have dramatic effects on gene activity (de Bono and Bargmann 1998; Glauser *et al.* 2011), we focused on high-impact variants as that resulted in a more manageable list of candidates. Assessment of linkage disequilibrium indicated that alleles within these two loci may not segregate randomly (Figure S5A), an effect that may be due to the genes being linked, or possibly due to some other population genetics-based-mechanisms; further investigation would be necessary to distinguish the reason for the disequilibrium. The breakdown of the genes within the QTL can be found in the Tables S6 and S7. A QTL was identified on chromosome V early in our analysis but was eliminated after the inclusion of all 66 strains. This QTL has been included as Table S8, since a few genes from this QTL were evaluated further. Additionally, for the QTL on chromosomes II and IV, the phenotype-by-genotype plots for peak markers within each QTL are shown in the supplementary materials (Figure S9).

### Pathways mediating attraction to prenol and IAA may be separable

IAA and prenol have similar chemical structures (Figure 2B) and both elicit attraction of N2 (wild-type control strain) (Figures 2 and 4). Among the wild isolates, the highest-scoring strain (JU1242), N2, and the set of strains that exhibited significantly reduced attraction to prenol (CB4856, LKC34, ECA191, JU775, and JU258) (Figure 3A) were evaluated for their responses to IAA (Figure 4) to determine whether natural variation among strains would result in different but specific responses to prenol and IAA. We found that CB4856 and ECA191 displayed reduced attraction to both odors, while LKC34, JU775, and JU258 exhibited much higher attraction to IAA compared to prenol (Figure 4). In addition, CB4856, ECA191, JU258, and JU775 all had reduced attraction to prenol compared to N2, while LKC34 and JU1242 responses were not statistically different compared to N2 (Figure S10). These data indicate that although there may be overlap in sensing prenol and IAA through the AWC neuron, there are likely genetic components (perhaps within the two identified QTL) that allow the differentiation of prenol and IAA.

### Multiple genes influence the response of *C. elegans* to prenol

Of the 95 gene candidates, 23 genes were investigated through evaluation of *lf* mutants. We also tested an additional four candidate-associated genes (genes that were commonly listed as associated genes for multiple gene candidates identified from *Ce*NDR results): *pgl-1**(**bn101**)*, *clk-1**(**qm30**)*, *mir-34**(**gk437**)* (which were associated with many of the high-impact candidates identified), and *dcap-1**(**ok2139**)*, a paralogue of *dcap-2* (Figure 5 and Figure S11). Among the *lf* mutants tested, mutants of three genes exhibited significantly reduced attraction to prenol compared to the N2 background: *dcap-2* (*ok2029* and *tm2470*), *dod-17* (*ok2387*), and *dcap-1* [*dcap-1*&Y55F3AM.13 (*ok2139*)].

To evaluate the specificity of each gene’s influence on the response to prenol, we tested the known *C. elegans* attractant IAA, which is structurally similar to prenol (Bargmann *et al.* 1993) (Figure 2A). We found that *dcap-2* (*lf*) and *dcap-1**(lf)* mutants exhibited reduced attraction to both prenol and IAA compared to the N2 wild type (*i.e.*, the reduced attraction is not specific to prenol). Interestingly, the *dod-17* (*ok2387*) (*lf*) mutant, which exhibited reduced attraction to prenol, appeared to have no defect in its attraction to IAA, as responses to IAA were comparable to those of the N2 wild-type control. However, testing additional *dod-17* (*lf*) alleles revealed that *dod-17* itself is not the cause of this effect (Figure S12). We also tested *lf* mutants of *rsd-2* (*tm1429*) (RNA interference spreading defective), which displayed the fourth lowest chemotactic response to prenol among the mutants tested; however, this mutant’s responses to prenol were not significantly different from those of N2 (Figure 5A). Surprisingly, the *rsd-2* (tm1429) *lf* mutant displayed significantly reduced attraction to IAA compared to N2 (Figure 5B). This trend was also mirrored by the missense mutation found in the *rsd-2* mutant, variant (*pk3307*) (see Figure S13). Lastly, we created rescue lines for the *dcap-2* (*lf*) allele *ok2023*. The rescue successfully improved attraction responses and demonstrated significantly higher responses than the (lf) mutant responses for prenol (Figure 5C). Similarly, responses to IAA were also significantly higher, but were still significantly less than the attraction observed in N2 (Figure 5C). Responses by individual rescue lines have also been detailed and provided in Figure S14.

*clec-39* (*lf*) did not have a reduced quadrant chemotaxis index to prenol (Figure 5A); however, the behavior exhibited by this strain was strikingly different from other mutants tested. Over 50% of the behavioral assays failed and additional assays were done to collect enough data for *clec-39* (*lf*) as the assays often did not meet the threshold of 50 nematodes (minimum) moving into one of the four quadrants of the plate during the allotted assay time. Representative videos are been provided in the supplementary materials [*clec-39* (*lf*) in Video S15 and N2 in Video S16]. It should be noted that the lack of nematodes moving out of the center for *clec-39* (*lf*) was observed even after the initial water from nematode placement is removed, thus water tension does not explain the lack of movement or participation in the assay. The phenotype was visually striking and was further explored. Participation of *clec-39* (*lf*) was compared to the wild-type control N2 using the standard chemotaxis assay (Baiocchi *et al.* 2017). The SCI values of *clec-39* (*lf*) were comparable to those of N2, but there was a large disparity in the participation observed between the two strains (Figure 6). The *clec-39* (*lf*) mutants exhibited significantly less participation, with nearly 80% of the nematodes remaining in the center of the assay arena compared to <50% of N2 nematodes remaining in the middle. This means that the SCI is only representative of ∼20% of the population for *clec-39** (lf)* mutants and suggests that *clec-39* affects nematode participation in foraging behavior. The function of *clec-39* and its involvement with this behavioral shift is not known, although it did not appear to cause any uncoordinated phenotype in the mutants. Additional testing using standard behavioral drop assays and touch assays were performed on this strain to further evaluate motility and found that motility and touch responsiveness and aversion behaviors were not different from N2 (neither were the touch responses and avoidance behaviors of *dod-17*, *dcap-2*, and *dcap-1* (lf) mutants) (Figure S17).

## Discussion

Prenol is associated with nematode-infected insects, suggesting that is an ecologically relevant odor and one that may be encountered by soil-dwelling organisms. Prenol elicits behavioral responses from a variety of nematodes including EPNs in the genus *Steinernema*, *L. texanum* (free-living nematode), *C. elegans*, *P. pacificus* (Figure S1), and even *Drosophila melanogaster* larvae (Baiocchi *et al.* 2017). It appears that both *C. elegans* dauers and young adults respond more strongly to prenol—especially at doses of 200 µM or below—compared to most EPN IJs (which only show distinct responses to doses at or above 20 mM) (Baiocchi *et al.* 2017; Kin *et al.* 2019). However, more evidence regarding the role of prenol in *C. elegans* ecology is still needed and the present study focused on how *C. elegans* responds to this odorant.

The AWC neurons are involved in the detection and response to numerous attractive odors, including IAA, 2-butanone, and benzaldehyde (Bargmann *et al.* 1993). Using genetically ablated neuron lines [ASI− (PY7505 *oyIs84*) and AWC− (PY7502 *oyIs85*)] (Figure 2A) and several strains with loss of function in neuron-associated genes (Figure 2C), we determined that the AWC neurons, and several canonical signaling components that are necessary for detecting other attractive odors, are required for detection and response to prenol. We also noted that while removal of the ASI neurons did not affect response to prenol, it did appear to affect response to IAA. Although the ASI neurons are involved in chemotaxis to several complex molecules (Bargmann and Horvitz 1991; Yoshida *et al.* 2012), the response to prenol and IAA by ASI-ablated mutants had not previously been evaluated in detail. It was known that the AWC neurons are required for adequate detection and response to IAA (Bargmann *et al.* 1993), although the data shown here suggest that ASI neurons may also provide a minor contribution to responses to IAA. Although these findings provide initial insights to the neuronal requirements for detecting prenol, further research would provide greater resolution and clarity. For instance, our experiments did not address the effects of molecular asymmetry regarding AWC-on and AWC-off and how these differences may be related to the detection and discrimination of prenol as they are in regard to other odors (Hsieh *et al.* 2014). This may be an important avenue of investigation considering that *str-2*, which encodes a g protein-coupled olfactory receptor, affected the response to IAA but not prenol, and thus may be directly involved in differentiating between similarly structured odors (Wes and Bargmann 2001). Additionally, future research in which a larger number of sensory neurons are evaluated would provide a great deal of clarity into the finer intricacies of odor detection, discrimination, and response.

The involvement of *odr-3*, *odr-1*, *tax-2*, *tax-4*, and *daf-11* was also shown through behavioral analyses, supporting previous studies suggesting that these genes (and their resulting proteins) are part of the genetic pathway necessary for AWC-mediated responses to attractive odorants (Bargmann 2006). Both *odr-1* and *daf-11* encode transmembrane guanylyl cyclases, with *odr-1* being involved in discrimination and adaptation (L’Etoile and Bargmann 2000) while *daf-11* is involved with multiple areas of chemosensation as well as dauer formation and recovery (Birnby *et al.* 2000; L’Etoile and Bargmann 2000). *odr-3* encodes a G-protein α-subunit, which has been shown to be involved with olfaction and nociception as well as aiding in morphogenesis of olfactory neurons in *C. elegans* (Roayaie *et al.* 1998). Lastly, *tax-2* and *tax-4* both encode subunits of a cyclic nucleotide gated channel that plays a key role in several aspects of *C. elegans* sensory abilities, including chemosensation, thermosensation, and even the formation of sensory neurons (Coburn *et al.* 1998). Our findings suggest that the AWC neurons and related genes are involved in the detection of prenol, and this supports our conclusion that prenol likely relies on a similar molecular pathway as other odorants. However, the exact molecular machinery that plays a role in initial detection, such as a specific GPCR, remains to be determined. Our results indicate that there are some distinct differences in the AWC mediated pathway for detecting and responding to prenol *vs.* the pathway for detecting and responding to IAA. We observed significant loss of attraction to IAA for many of loss-of-function mutants evaluated, where the response to prenol was not similarly affected. Furthermore, for *lf* strains of *gpa-2*, *str-2*, and *odr-4* we found that the attraction to prenol was significantly higher than the attraction to IAA. These results suggest some of the machinery involved in the ability of *C. elegans* to discriminate between odors that activate the same neuron.

The finding that some natural isolates differed in their responses to prenol *vs.* IAA (Figure 4) indicated that response to these odors is genetically separable, and that there could be a discernable genetic component within the QTL we identified. Evaluation of candidate genes resulted in identification of four genes that, when knocked out, appeared to reduce attraction to prenol, none of which had previously been implicated in any chemotaxis behavior. *clec-39* was known to be involved in immune responses to *Serratia marcescens* and although C-type lectins (CLECs) can regulate immune responses both at the physiological level and behavioral level (Pees *et al.* 2017), it is not known how or why *clec-39* (*lf*) results in significantly reduced participation in chemotaxis. One potential explanation is that the loss of *clec-39* may influence social behaviors, not unlike the shift to social feeding behavior when *npr-1* is altered in a specific way (de Bono and Bargmann 1998). There is evidence to suggest that some *clec* genes (and their protein products) such as *clec-164* and *clec-179* are not only produced in neurons but are involved with extracellular vessel secretion and modulation of behaviors (Wang *et al.* 2015). Currently, not much is known about *clec-39* except for suggestions about its potential involvement in immunity. However, it is possible that it may be involved in aggregation or clumping behaviors, but more investigation is needed to elucidate the exact involvement of *clec-39* with this behavioral phenotype.

The connection between *dcap-2* and its paralog *dcap-1* to behavior is a bit clearer than that of *clec-39*. Although these genes were not previously implicated directly in attraction behavior, it was known that these two genes are needed for the proper formation of the AWC sensory cilia (Adachi *et al.* 2017). Furthermore, *dcap-1* and *dcap-2* exhibit similar levels of reduced attraction to IAA, indicating that their roles in chemosensation are not specific to prenol.

Among the genes identified, *dod-17* initially stood out as having a specific effect on response to prenol but not IAA (Figure 5B). *dod-17* is expressed mainly in the intestine and is predicted to be involved in innate immunity (Table S18), but the exact function of *dod-17* remains unknown. However, additional (lf) alleles created to investigate *dod-17* did not support the initial results, suggesting that there are other background mutations in the RB1845 CGC strain (*ok2387**)* that might account for the observed shift in phenotype; additional research would be needed to identify the cause.

We have established AWC neuron involvement in detecting prenol and we have identified several genes that affect the response to prenol. We wondered whether examining the transcriptional profile of genes with high-impact genetic variants that we identified would reveal any genes of interest (Hsueh *et al.* 2017) (Figure S19). The transcriptional data revealed that among *dcap-1*, *dcap-2*, *clec*-39, and *dod-17*, none had differential expression between the AWC and whole larvae, with the exception of *dcap-1*, which had significantly lower expression in the AWC. In addition, several of the genes we tested that bore no influence on response to prenol also had lower expression in the AWC (Figure S19). One caveat is that our comparisons come from two very different populations. It is worth noting that the transcriptional data were sourced from mixed populations and thus the transcriptional data may not be a perfect representation of the population that has been the focus within this study (the young adult stage). A comparison of these resources still provides helpful information in limiting the gene candidate lists to potentially unveil other genes that might be involved with detection and response to prenol. For instance, three genes that contained high-impact variants in our study appear to have significantly higher expression in AWC compared to whole larvae: *ttr-51*, *Y45F10D.7*, and *rsd-2*. There were also two other genes with >10-fold higher expression in the AWC neuron but that were not found to be statistically significantly differentially expressed: *cul-6* and *F09E8.2*. These may be good candidates for future studies of AWC-mediated behavior.

Information about particular genes, such as those that play critical roles in the AWC neurons, has been used in previous studies to provide a better understanding of the molecular underpinnings of free-living and parasitic nematode behavior. For genes identified in this study, which affect response to prenol, several have homologs in EPNs and in nematode parasites of mammals (Figure S2), including *dcap-1*, *dcap-2*, and *clec-39* in addition to the AWC-related genes *odr-1*, *odr-3*, *daf-11*, *tax-2*, and *tax-4*. Such information is of value not just for the improved understanding of *C. elegans* behavioral genetics, but also for understanding what types of genes might be implicated in the detection of olfactory cues in other species of nematodes, such as the EPNs with which the connection between prenol and nematodes was first made. While the molecular genetic pathway underpinning response to prenol is not fully understood, the work reported here provides a foundation for future evaluation and efforts to bridge the gap between *C. elegans* biology and its application in EPNs.
